# Supplementary figures and images for: A systematic review and narrative synthesis of the research provisions under the Mental Capacity Act (2005) in England and Wales: Recruitment of adults with capacity and communication difficulties
Source: PLoS One. 2021 Sep 1;16(9):e0256697. doi: 10.1371/journal.pone.0256697 (PMC8409627; doi:10.1371/journal.pone.0256697)

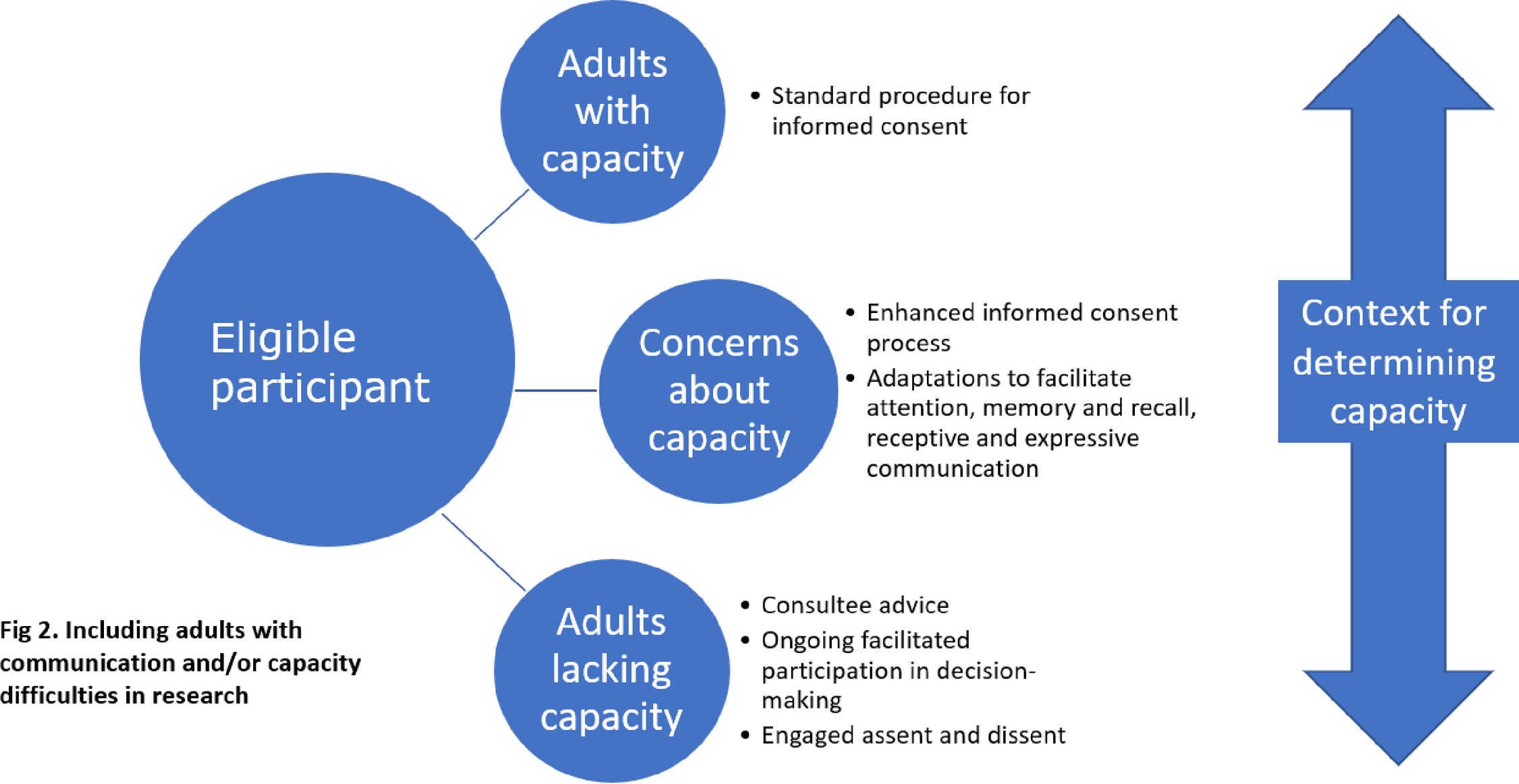

Supplement: S1 Fig — (TIF) [file pone.0256697.s001.tif]
