# Supplementary material for: A systematic review and narrative synthesis of the research provisions under the Mental Capacity Act (2005) in England and Wales: Recruitment of adults with capacity and communication difficulties
Source: PLoS One. 2021 Sep 1;16(9):e0256697. doi: 10.1371/journal.pone.0256697 (PMC8409627; doi:10.1371/journal.pone.0256697)
Supplement: S3 Table — (DOCX) [file pone.0256697.s004.docx]

**S5 Table. Characteristics of included studies**

|  | **Article** | | | **Sample** | | | | **Recruitment** | | | | | | | **Accommo-dations**^[[1]](#footnote-1)^ | |
| --- | --- | --- | --- | --- | --- | --- | --- | --- | --- | --- | --- | --- | --- | --- | --- | --- |
|  | Author(s) | Year | Title & Journal | CCD | Type ^[[2]](#footnote-2)^ | Non-CCD | Inclusion ^[[3]](#footnote-3)^ | Gatekeeper/Significant other^[[4]](#footnote-4)^ | Inf. Consent  format ^[[5]](#footnote-5)^ | Info. format ^[[6]](#footnote-6)^ | Capacity Assess^[[7]](#footnote-7)^ | Consultee ^[[8]](#footnote-8)^ | Engage-ment ^[[9]](#footnote-9)^ |  | |  |
|  | Frighi, V et al. | 2011 | Safety of antipsychotics in people with intellectual disability. *The British Journal of Psychiatry 199: 289–295* | 202 | ID | 9000+ | SR, Sy | NHS Trust Clinicians | W | NR | As in MCA/CoP | P and/or N | A | FP | |  |
|  | Goldsmith, L. et al | 2013 | Informed consent for blood tests in people with a learning disability. *Journal of advanced nursing 69(9), 1966–1976* | 14 | ID | 0 | C, Sy | LD nurses, care workers, support staff (community and GP) | O + P | AF | DNP | No | A/D | SO, AP | |  |
|  | Lloyd LC. et al | 2013 | Service users’ experiences, understanding and hopes about care in an inpatient intellectual disability unit: a qualitative study. *Advances in mental health and intellectual disabilities 7(4): 201-210* | 8 | ID | 0 | Sy, I-P,  Co | NHS trust | DNP | ST, V | DNP | No | D | AP | |  |
|  | Frighi, V et al. | 2014 | Vitamin D deficiency in patients with intellectual disabilities: prevalence, risk factors and management strategies  *The British Journal of Psychiatry 205: 458–464* | 155 | ID | 192 | SR, Sy | NHS Trust Clinicians | W | NR | As in MCA/CoP | P and/or N | A | FP | |  |
|  | Inchley-Mort, S. et al | 2014a | Complex Behaviour Service: enhanced model for challenging behaviour. *Advances in mental health and intellectual disabilities 8 (4): 219-227* | 24 | ID | 22 | SR | Service register | W | NR | DNP | N | NR | NR | |  |
|  | Inchley-Mort, S. et al | 2014b | Complex Behaviour Service: content analysis of stakeholder opinions. *Advances in mental health and intellectual disabilities 8 (4): 228-236* | 6 | ID | 25 | SR, CO | Service register | W | NR | DNP | No | NR | NR | |  |
|  | Jayes, M. & Palmer, R | 2014 | Initial evaluation of the Consent Support Tool: A structured procedure to facilitate the inclusion and engagement of people with aphasia in the informed consent process  *International Journal of Speech-Language Pathology, 16(2): 159–168* | 14 | Aph | 0 | SR | Ongoing study | W | AF, V | Closed questions with multiple choice responses | P | A | FL  Aphasia friendly consent form | |  |
|  | Sampson, E. L. et al | 2014 | Behavioural and psychiatric symptoms in people with dementia admitted to the acute hospital: prospective cohort study. *The British Journal of Psychiatry 205: 189–196.* | 230 | De | 0 | SR, Sy, Eng, I-P | NHS Trust | W | NR | Structured assessment | P/N | A | FL | |  |
|  | Godwin, B. & Poland, F. | 2015 | Bedlam or bliss? Recognising the emotional self-experience of people with moderate to advanced dementia in residential and nursing care. *Quality in ageing and older adults 16 (4): 235-248* | 13 | De | 0 | Sy, CO | Care home manager | DNP | NR | DNP | No | A/D | NR | |  |
|  | Hughes, T. & Romero, M.C. | 2015 | A procedural consent methodology with people diagnosed with dementia  *Quality in ageing and older adults 16(4): 222-234* | 8 | De | 0 | C, Eng, Sy | Adult social care service (staff) | O + P | AF, V | Behaviour checked reflexively for understanding and consent. | No | A/D | AP, CP | |  |
|  | Khalifeh, H. et al | 2015 | Violent and non-violent crime against adults with severe mental illness. *The British Journal of Psychiatry 206: 275–282* | 361 | MH | 3138 | Sy, C, Eng, SR | Care coordinators | W | ST | DNP | No | A | NR | |  |
|  | McCarthy, J. et al | 2015 | Screening and diagnostic assessment of neurodevelopmental disorders in a male prison. *Journal of intellectual disabilities and offending behaviour 6* *(2): 102-111,* | 240 | ADHD, ASD & ID | 0 | C, Eng, | Prison staff. | W | ET, V | checklist – the four-capacity basis. | No | NR | AP, PR, TI | |  |
|  | Robotham, D. et al | 2015 | Linking a research register to clinical records in older adults’ mental health services: a mixed-methods study. *Alzheimer's Research & Therapy 7:15-21* | 31 | De | 37 | SR, C | NHS Trust | DNP | ST | DNP | No | NR | NR | |  |
|  | Sampson, E. L. et al | 2015 | Pain, agitation, and behavioural problems in people with dementia admitted to general hospital wards: a longitudinal cohort study. *Pain J. 156 (4): 765-683.* | 230 | De | 0 | SR, Sy, Eng | Clinical staff | W | NR | DNP | P/N | A | FL | |  |
|  | Brugha, T.S. et al | 2016 | Epidemiology of autism in adults across age groups and ability levels. *The British Journal of Psychiatry 209: 498–503* | 217 | ID | 0 | Sy; SR | Study register | DNP | ST | DNP | AP | NR | FL | |  |
|  | Larson, F.V. et al | 2017 | Psychosis in autism: comparison of the features of both conditions in a dually affected cohort. *The British Journal of Psychiatry 210: 269–275* | 116 | ASD/MH | 568 | Sy | Clinicians in services across UK, charities and self-referral | W | NR | DNP | P | NR | NR | |  |
|  | Malik, K. J. et al | 2017 | The complex role of social care services in supporting the development of sustainable identities: Insights from the experiences of British South Asian women with intellectual disabilities. *Research in Developmental Disabilities 63: 74–84* | 10 | ID | 0 | Sy, C | Education, social care, local authority, charities, private service providers and health services (seven organisations) | DNP | NR | DNP | No | NR | TI | |  |
|  | Durling, E. et al | 2018 | Family and community in the lives of UK Bangladeshi parents with intellectual disabilities. *J Appl Res Intellect Disabil. 31:1133–1143* | 4 | ID | 10 | C, SR | Community Learning Disability Service | DNP | ER | Not described | No | NR | NR | |  |
|  | Feast A.R. et al | 2018 | Pain and delirium in people with dementia in the acute general hospital setting*. Age and Ageing 2018; 47: 841–846.* | 230 | De | 0 | Sy, Co, I-P | NHS trust  Clinicians (geriatricians) | W | NR | Based on MCA | P/N | A/D | NR | |  |
|  | Larson F.V. et al | 2018 | Copy number variants in people with autism spectrum disorders and comorbid psychosis*. European Journal of Medical Genetics 61: 230–234.* | 116 | ASD & Psychosis | 0 | Sy | Public and independent clinical services, charities, ASD social networking website. | DNP | NR | DNP. | AP | NR | NR | |  |
|  | Spencer, B.W.J. Et al | 2018 | Unwell in hospital but not incapable: cross sectional study on the dissociation of decision making capacity for treatment and research in in-patients with schizophrenia and related psychoses. *The British Journal of Psychiatry 213: 484–489* | 84 | MH | 0 | Sy, Eng,  I-P | NHS Trust | DNP | ST | Semi-structured interview.  MacArthur Competence Assessment Tool for Clinical Research | N | NR | CP | |  |
|  | Fish, R. & Morgan, H. | 2019 | Moving on” through the locked ward system for women with  intellectual disabilities. *J Appl Res Intellect Disabil. 32:932–941.* | 16 | ID | 10 | Sy, C | NHS secure unit  Ward managers | W | ER, AF, V | Understanding checked according to MCA | No | NR | NR | |  |
|  | Frighi, V. et al | 2019 | Vitamin D, bone mineral density and risk of fracture in people with intellectual disabilities. Journal of *Intellectual Disability Research 63(4) 357–367.* | 51 | ID | 0 | Sy, SR | NHS Trust Clinicians | W | ER | Understanding checked according to MCA | P/N | NR | SO, FP | |  |
|  | Hall, A., et al | 2019 | Moving beyond ‘safety’ versus ‘autonomy’: a qualitative exploration of the ethics of using monitoring technologies in long-term dementia care. *BMC Geriatrics 19:145.* | 3 | De | 33 | Sy | Care home staff | O + P | ST, V | Informal -  guidance from staff and family members | P | A/D | AP | |  |
|  | Kelley,R., et al | 2019 | The impacts of family involvement on general hospital care experiences for people living with dementia: An ethnographic study. *International Journal of Nursing Studies 96 : 72–81* | 12 | De | 34 | Sy, Eng,  I-P | NHS trust  Nursing staff | W | PO, V | Informal –  assessed during conversations | P | A/D | NR | |  |
|  | Sheehan, R. et al | 2019 | Experiences of psychotropic medication use and decision- making for adults with intellectual disability: a multistake holder qualitative study in the UK. *BMJ Open 2019;9:e032861* | 14 | ID | 24 | Sy, SR | NHS Trust  Clinicians  Direct link during presentation | W | ST, ER, V | Assessed according to MCA | No | NR | CP, PR | |  |
|  | Stoner, CR., | 2019 | The psychometric properties of the control, autonomy, self-realisation and pleasure scale (CASP-19) for older adults with dementia. *Aging & Mental Health, 23:5, 643-649* | 225 | De | 0 | Sy, C | Study register  Research assistant within NHS trust | W | ST, AF, ER | Informal capacity assessment | NR | NR | NR | |  |
|  | Wray, F. et al | 2019 | How do stroke survivors with communication difficulties manage life after stroke in the first year? A qualitative study. *International Journal of Language and Communication Disorders, 54 (5). pp. 814-827* | 14 | Aph | 7 | SR, C | NHS trust  (hospital and community-based services)  Staff (SLT) | DNP | V | Informal - use of the Consent Support Tool | NR | NR | AP, AT, PR | |  |

1. Coproduction = CP; Familiar place = FP; Supporter option = SO; Adapted tools = AT; Translator = TR; Interpreter = IP; Adapted process = AP; Flexibility = FL; Verbal cues =VC; Visual prompt = VP; Privacy = P; AIO = Accessible Information Officer; PR = Privacy; TI = Time; PR = Professional experienced/training; Not reported = NR [↑](#footnote-ref-1)
2. Intellectual disability = ID; Autistic Spectrum Disorder = ASD; Dementia = De; Aphasia post-stroke = Aph; Acquired Brain Injury = ABI; Mental Health = MH; Physical disability = PD [↑](#footnote-ref-2)
3. Capacity = C; Symptomatology = Sy; Service Register = SR; Sensory function = SF; English speaking = Eng; I-P =In-patient; Co = Communication skills [↑](#footnote-ref-3)
4. Significant Other/Gatekeeper: Named [↑](#footnote-ref-4)
5. Informed Consent: Written = W; Oral consent as part of an interactive process = O + P, Details not provided = DNP [↑](#footnote-ref-5)
6. Standard text/Written – ST; Easy text = ET (simplified text); Easy read (text with pictures) = ER; Verbal = V; Picture Cards =PC; Accessible format = AF; Poster = PO [↑](#footnote-ref-6)
7. Capacity assessment: Yes = Y; No = N; Details not provided = DNP [↑](#footnote-ref-7)
8. Consultee: Personal = P; Nominated = N; AP = Appropriate [↑](#footnote-ref-8)
9. Engagement = Assent = A; Dissent = D [↑](#footnote-ref-9)
