# Supplementary material for: A systematic review and narrative synthesis of the research provisions under the Mental Capacity Act (2005) in England and Wales: Recruitment of adults with capacity and communication difficulties
Source: PLoS One. 2021 Sep 1;16(9):e0256697. doi: 10.1371/journal.pone.0256697 (PMC8409627; doi:10.1371/journal.pone.0256697)
Supplement: S5 Table — (DOCX) [file pone.0256697.s006.docx]

**S7. Table. Summary table of study synopses (Studies 1-15) MMAT**

| 1. Frighi et al 2011 | Clear description of the target population, the variables are clearly defined and accurately measured, validated and reliable tested measures, all participants accounted for, 6% exclusions from results, logistic regression model used to adjust for risk factors. metabolic and anthropometric data from participants with diabetes on hypoglycaemic therapy were not included in the comparisons of the antipsychotic-treated v. antipsychotic naive groups. |
| --- | --- |
| 2. Goldsmith et al 2013 | Qualitative method - ethnography appropriate as it explored the knowledge and attitudes of people with learning disability. Focused ethnography has a shorter timescale than traditional ethnographies, which might be useful in people with LD. Data substantiated with quotes with clear links between data sources, analysis and interpretation. |
| 3. Lloyd et al 2013 | Qualitative method using grounded theory was appropriate as it creates an open approach to exploring service users’ experiences. Semi-structured method of collecting data, results substantiated with data in the form of quotes. Coherence between data and its interpretation. |
| 4. Frighi et al 2014 | Quantitative non-randomized studies, which used cross-sectional observational method, clear description of the target population, the variables are clearly defined and accurately measured, validated and reliability tested measures, all participants accounted for, 6% exclusions from final results, logistic regression model used to adjust for risk factors. metabolic and anthropometric data from participants with diabetes on hypoglycaemic therapy were not included in the comparisons of the antipsychotic-treated v. antipsychotic naive groups |
| 5. Inchley-Mort et al 2014a | Quantitative non-randomized studies, cohort study which followed participants for over a year. Participants were representative of target population (service users treated by the CBS), measurements used were validated measures. Analysis was not adjusted for multiple testing - source of bias for the results. All 46 individuals had access to medical/psychiatric, nursing, psychological or other interventions including social care as part of the usual care provision, so no difference in access to support. |
| 6. Inchley-Mort, et al 2014b | Qualitative method is appropriate for obtaining opinions and experiences, semi-structured interview adequate for those with intellectual disabilities. However, it was convenience sampling and those with more profound disability were excluded, it may therefore not be representative. Quotes from data to support results and the interpretation of it. We acknowledge limitations relating to interviewees having felt potentially unable to be explicitly critical of a service. a service user spoke freely prior to the formally recorded interview but he spoke less and answered “I don’t know” to questions that he had previously answered differently once the recording of the interview had begun. |
| 7. Jayes, M. and Palmer 2014 | Mixed method. Case series (Quantitative descriptive studies) and qualitative (semi-structured interview), mixed methods design. Qualitive approach was appropriate for evaluating the CST - obtaining views of users which used semi-structure interview, data was substantiated with quotes, with clear links between data and its interpretation. Convenience sampling, all individuals expressing an interest were recruited, very small number (n=14), it was not clear how many participants were interviewed. there was no way to compared respondence to non-responders. small sample size used is likely to have limited its representativeness and thus compromised both the generalizability of the results and the precision of the estimated statistical level of agreement |
| 8. Sampson et al 2014 | Quantitative non-randomized studies, longitudinal cohort study design , large number of participants, sample size calculated, ,measures were based on validated tools, flow of participants reported , e.g. the use of DSM criteria, appropriate statistical methods such as linear and logistic regressions as well as sensitivity analysis. |
| 9. Godwin & Poland 2015 | Qualitative. The use of Interpretative Phenomenological Analysis approach in people living with dementia can help to provide detailed examination of their personal lived experience. Use of semi-structured interview and observation of verbal and non-verbal behaviour - could provide multiple perspective, results substantiated with data and discussed well. |
| 10. Hughes & Romero 2015 | Qualitative methods right, data collection: conversations and one-to-one interviews; multiple methods allowing for in depth understanding, data was used to support findings and interpretations were clearly linked with data sources. |
| 11. Khalifeh et al 2015 | Quantitative non-randomized studies aimed at estimating the prevalence and impact of crime among people with SMI compared with the general population. A cross-sectional study with representative large population, measures are appropriate for answering the research question, flow of participants reported, confounders accounted for in analysis - use of adjustments, logistic regression and sensitivity analysis. bias reduced with the use of structured questionnaire. |
| 12. McCarthy et al 2015 | Quantitative descriptive studies. purposive sampling strategy was used to maximise recruitment and increase representativeness; but bias is reflected by the recruitment of only those who were assessed as having capacity to give informed consent (see below) and could understand/speak sufficient English. measures were appropriate. statistical analysis as relevant. |
| 13. Robotham et al 2015 | A combination of cross-sectional observational study with explanatory interview used to capture different aspects of the service. Findings are derived from the data, which generated codes substantiated by quotes from participants. However, the quantitative component was less organised. there was no way to tell if there were sufficient participants, statistical analysis was minimal and there was no way to check if confounders were minimised. |
| 14. Sampson et al 2015 | Quantitative non-randomized cohort study. Sample drawn from two acute hospitals serving a population of two million from six primary care trusts and four mental health trusts in London, they were representative of target population which was people with dementia admitted to the acute hospital, sample size estimated, variables measured with reliable measures , flow of participants through the study presented, attrition is minimal, statistical analysis methods is adequate + sensitivity analysis carried out |
| 15. Brugha et al 2016 | Overall score is 4. Quantitative descriptive studies of the prevalence of autism and key associations in adults of all ages and abilities. Sample was drawn by random probability selection from two large registers, representative samples of those previous excluded when the registers were created. Measures were appropriate, variables clearly defined, diagnostic interviews conducted by experienced people who have received induction and training programme. Although the paper reported no evidence of non-response bias, selection bias was possible due to low response rate in the recruitment for one of the registers used. This was acknowledged as a weakness of the study. Statistical analysis was appropriate. Sensitivity analysis was conducted, measures of intellectual disability was standardized, and logistic regression used to adjust for age, gender and disability levels. |

**MMAT quality assessment summary table 1a (Studies 1-15)**

Key: 1 = Yes; 0 = No; - = Can’t tell; ∕not applicable

| **MMAT Items (1., 3.)** | **1** | **2** | **3** | **4** | **5** | **6** | **7** | **8** | **9** | **10** | **11** | **12** | **13** | **14** | **15** |
| --- | --- | --- | --- | --- | --- | --- | --- | --- | --- | --- | --- | --- | --- | --- | --- |
| **Screening questions** |  | | | | | | | | | | | | | | |
| S1. Clear research questions? | 1 | 1 | 1 | 1 | 1 | 1 | 1 | 1 | 1 | 1 | 1 | 1 | 1 | 1 | 1 |
| S2. Data allow address of research questions? | 1 | 1 | 1 | 1 | 1 | 1 | 1 | 1 | 1 | 1 | 1 | 1 | 1 | 1 | 1 |
| **1.QUALITATIVE STUDIES** |  | | | | | | | | | | | | | | |
| 1.1. Approach appropriate to answer research question? | **/** | **1** | **1** | **/** | **/** | **1** | **1** | **/** | **1** | **1** | **/** | **/** | **1** | **/** | **/** |
| 1.2. Data collection methods adequate to address research question? | **/** | **1** | **1** | **/** | **/** | **1** | **1** | **/** | **1** | **1** | **/** | **/** | **1** | **/** | **/** |
| 1.3. Findings adequately derived from the data? | **/** | **1** | **1** | **/** | **/** | **1** | **1** | **/** | **1** | **1** | **/** | **/** | **1** | **/** | **/** |
| 1.4. Is the interpretation of results sufficiently substantiated by data? | **/** | **1** | **1** | **/** | **/** | **-** | **1** | **/** | **1** | **1** | **/** | **/** | **1** | **/** | **/** |
| 1.5. Coherence between data sources, collection, analysis and interpretation? | **/** | **1** | **1** | **/** | **/** | **1** | **1** | **/** | **1** | **1** | **/** | **/** | **1** | **/** | **/** |
| **2. RANDOMIZED CONTROLLED TRIALS** |  | | | | | | | | | | | | | | |
| 2.1. Is randomization appropriately performed? | **/** | **/** | **/** | **/** | **/** | **/** | **/** | **/** | **/** | **/** | **/** | **/** | **/** | **/** | **/** |
| 2.2. Are the groups comparable at baseline? | **/** | **/** | **/** | **/** | **/** | **/** | **/** | **/** | **/** | **/** | **/** | **/** | **/** | **/** | **/** |
| 2.3. Are there complete outcome data? | **/** | **/** | **/** | **/** | **/** | **/** | **/** | **/** | **/** | **/** | **/** | **/** | **/** | **/** | **/** |
| 2.4. Are outcome assessors blinded to the intervention provided? | **/** | **/** | **/** | **/** | **/** | **/** | **/** | **/** | **/** | **/** | **/** | **/** | **/** | **/** | **/** |
| 2.5 Did the participants adhere to the assigned intervention? | **/** | **/** | **/** | **/** | **/** | **/** | **/** | **/** | **/** | **/** | **/** | **/** | **/** | **/** | **/** |
| **3. NON-RANDOMIZED STUDIES** |  | | | | | | | | | | | | | | |
| 3.1. Participants representative of target population? | **1** | **/** | **/** | **1** | **1** | **/** | **/** | **1** | **/** | **/** | **1** | **/** | **1** | **1** | **/** |
| 3.2. Measurements appropriate to both outcome and intervention/exposure? | **1** | **/** | **/** | **1** | **1** | **/** | **/** | **1** | **/** | **/** | **1** | **/** | **1** | **1** | **/** |
| 3.3. Complete outcome data? | **1** | **/** | **/** | **1** | **1** | **/** | **/** | **1** | **/** | **/** | **1** | **/** | **-** | **1** | **/** |
| 3.4. Confounders accounted for in design and analysis? | **1** | **/** | **/** | **1** | **0** | **/** | **/** | **1** | **/** | **/** | **1** | **/** | **-** | **1** | **/** |
| 3.5. During study period, intervention administered/exposure occurred as intended? | **1** | **/** | **/** | **1** | **1** | **/** | **/** | **1** | **/** | **/** | **1** | **/** | **1** | **1** | **/** |

| **MMAT Items (4., 5.)** | **1** | **2** | **3** | **4** | **5** | **6** | **7** | **8** | **9** | **10** | **11** | **12** | **13** | **14** | **15** |
| --- | --- | --- | --- | --- | --- | --- | --- | --- | --- | --- | --- | --- | --- | --- | --- |
| **4. QUANTITATIVE DESCRIPTIVE STUDIES** |  | | | | | | | | | | | | | | |
| 4.1. Sampling strategy relevant to research question? | **/** | **/** | **/** | **/** | **/** | **/** | **1** | **/** | **/** | **/** | **/** | **0** | **/** | **/** | **1** |
| 4.2. Sample representative of target population? | **/** | **/** | **/** | **/** | **/** | **/** | **-** | **/** | **/** | **/** | **/** | **0** | **/** | **/** | **1** |
| 4.3. Measurements appropriate? | **/** | **/** | **/** | **/** | **/** | **/** | **1** | **/** | **/** | **/** | **/** | **1** | **/** | **/** | **1** |
| 4.4. Risk of nonresponse bias low? | **/** | **/** | **/** | **/** | **/** | **/** | **0** | **/** | **/** | **/** | **/** | **-** | **/** | **/** | **1** |
| 4.5. Statistical analysis appropriate to answer research question? | **/** | **/** | **/** | **/** | **/** | **/** | **1** | **/** | **/** | **/** | **/** | **1** | **/** | **/** |  |
| **5. MIXED METHODS STUDIES** |  | | | | | | | | | | | | | | |
| 5.1. Adequate rationale for using mixed methods design to address research question? | **/** | **/** | **/** | **/** | **/** | **/** | **1** | **/** | **/** | **/** | **/** | **/** | **1** | **/** | **/** |
| 5.2. Different components of study effectively integrated to answer research question? | **/** | **/** | **/** | **/** | **/** | **/** | **1** | **/** | **/** | **/** | **/** | **/** | **1** | **/** | **/** |
| 5.3. Outputs of integration of qualitative and quantitative components adequately interpreted? | **/** | **/** | **/** | **/** | **/** | **/** | **1** | **/** | **/** | **/** | **/** | **/** | **1** | **/** | **/** |
| 5.4. Divergences and inconsistencies between quantitative and qualitative results adequately addressed? | **/** | **/** | **/** | **/** | **/** | **/** | **-** | **/** | **/** | **/** | **/** | **/** | **1** | **/** | **/** |
| 5.5. Different components of study adhere to quality criteria of each tradition of methods involved? | **/** | **/** | **/** | **/** | **/** | **/** | **1** | **/** | **/** | **/** | **/** | **/** | **1** | **/** | **/** |
| **Total score** | **5** | **5** | **5** | **5** | **4** | **4** | **3** | **5** | **5** | **5** | **5** | **2** | **3** | **5** | **5** |

**Summary table of study synopses (Studies 16-28) MMAT**

| 16. Larson et al 2017 | Quantitative non-randomized cohort study. large sample size not based on calculation but time allowed for recruitment, was not systematic but inclusion criteria clearly defined, use of appropriate measures, control were obtained from previously published data, methods used for analysis were less robust than they would have been with a well-matched sample. regression models used and a smaller p-value - 0.017. |
| --- | --- |
| 17. Malik et al 2017 | Qualitative methods (IPA) ideal for exploring experiences, findings are arranged into themes and subthemes, substantiated with quotes from participants. Clear links between data and interpretation |
| 18. Durling et al 2018 | Qualitative method is appropriate for the research question, interview plus field notes used for data collection. Thematic analysis was carried (method for identifying, analysing and reporting patterns (themes) within data) - verbatim transcription of interviews. Where an interpreter was used, the interpreter listened to audio- recordings and checked transcripts both for accuracy of transcription and accuracy of translation, correcting where necessary. Results derived from data - themes and subthemes, supported by quotes from participants. Data sources, analysis and interpretations were linked. |
| 19. Feast et al 2018 | Quantitative non-randomized studies; observational prospective longitudinal cohort study. Sample size determined, inclusion, exclusion criteria stated and reasons for excluding some participants were stated e.g. delirium. measurements are justified, logistic regressions, All analyses were adjusted for potential confounders (age, dementia severity, BPSD and Charlson comorbidity scores) |
| 20. Larson et al 2018 | Quantitative non-randomized studies. Large sample size was not based on calculation but on time allowed for recruitment, but inclusion criteria clearly defined, use of appropriate measures, control were obtained from published data, regression models used and a smaller p-value - 0.017. |
| 21. Spencer et al 2018 | Quantitative non-randomized studies, cross-sectional study. Inclusion, exclusion criteria provided, reasons for exclusion provided. Measurements are justified for outcome variables and based on validated measures, over 70% of missing cases on an item, to compensate for this, they restricted the analysis of PANSS and neurocognitive items for cases in which we had full data, so that direct comparisons of symptoms and associations with DMC-R and DMC-T could be made. Statistical analysis methods such as calculation of z-scores, trichomising skewed scores and using data on a 'complete case' analysis allowed direct comparison of measures. |
| 22. Fish & Morgan 2019 | Qualitative, method used was ethnographic study - field‐notes and semi‐structured interviews - appropriate to observe and study rea-life environment. Findings are derived from data - analysis was inductive, arising from the data, substantiated by quotes. There are clear links between data and interpretation of it. |
| 23. Frighi et al 2019 | Quantitative non-randomized studies, which used observational, cohort-based study method. Participants were representative of the target population - those diagnosed with vitamin D deficiency. Inclusion criteria clear and those not recruited were accounted for. Measurements were made using validated methods, no drop out, t-scores and z-scores were calculated for measures. sample size was large enough for univariate analyses but too small for regression analysis |
| 24. Hall et al 2019 | Qualitative study. The use of observation and semi-structured interview allowed the researcher to document the experiences of participants as it happens. Codes and themes derived from data and substantiated by quotes from participants. Links between data sources and interpretation |
| 25. Kelley et al 2019 | Qualitative study. A study aimed to explore how family involvement impacts on the experiences of hospital care for people living with dementia used ethnographic method - observations (400 hours), conversations and interviews. Data collection methods adequate to address research questions. in depth data analysis - ground theory, comparative approach to integrate data collection and analysis., followed by identification of codes and themes. this was substantiated with quotes from participants and discussed |
| 26. Sheehan et al 2019 | Qualitative study. thematic analysis allowed inductive orientation which allowed themes to be derived from the data. Purposive sampling was used to select participants with a range of characteristics that may be related to medication views and experiences. In-depth semi-structured interviews that were based on participants ability. Interview topic guide allowed points of interests to be pursued as they arose. Themes derived from data, substantiated by quotes. |
| 27. Stoner et al 2019 | Quantitative non-randomized studies, observational study with sample drawn strategically to include target population of older adults with dementia from five NHS trusts across England. Inclusion and exclusion criteria were described, measurements were validated. There was no way to judge non-response bias. Statistical analysis methods catered for missing data and data were standardised. |
| 28. Wray et al 2019 | Qualitative method is appropriate as the research aimed to explore how stroke survivors with communication difficulties and their family members manage life after stroke. semi-structured interviews and thematic analysis were employed. Data collection methods were adapted for the group - supported conversation techniques, topic guide used flexibly, sorting card with Talking Mats. Results substantiated with quotes from participants and key themes discussed. |

**MMAT quality assessment summary table 1b (Studies 16-28)**

Key: 1 = Yes; 0 = No; - =can’t tell; ∕not applicable

| **MMAT Items (1., 3.)** | **16** | **17** | **18** | **19** | **20** | **21** | **22** | **23** | **24** | **25** | **26** | **27** | **28** |
| --- | --- | --- | --- | --- | --- | --- | --- | --- | --- | --- | --- | --- | --- |
| **Screening questions** |  | | | | | | | | | | | | |
| S1. Clear research questions? | 1 | 1 | 1 | 1 | 1 | 1 | 1 | 1 | 1 | 1 | 1 | 1 | 1 |
| S2. Data allow address of research questions? | 1 | 1 | 1 | 1 | 1 | 1 | 1 | 1 | 1 | 1 | 1 | 1 | 1 |
| **1.QUALITATIVE STUDIES** |  | | | | | | | | | | | | |
| 1.1. Approach appropriate to answer research question? | **/** | **1** | **1** | **/** | **/** | **/** | **1** | **/** | **1** | **1** | **1** | **/** | **1** |
| 1.2. Data collection methods adequate to address research question? | **/** | **1** | **1** | **/** | **/** | **/** | **1** | **/** | **1** | **1** | **1** | **/** | **1** |
| 1.3. Findings adequately derived from the data? | **/** | **1** | **1** | **/** | **/** | **/** | **1** | **/** | **1** | **1** | **1** | **/** | **1** |
| 1.4. Is the interpretation of results sufficiently substantiated by data? | **/** | **1** | **1** | **/** | **/** | **/** | **1** | **/** | **1** | **1** | **1** | **/** | **1** |
| 1.5. Coherence between data sources, collection, analysis and interpretation? | **/** | **1** | **1** | **/** | **/** | **/** | **1** | **/** | **1** | **1** | **1** | **/** | **1** |
| **2. RANDOMIZED CONTROLLED TRIALS** |  | | | | | | | | | | | | |
| 2.1. Is randomization appropriately performed? | **/** | **/** | **/** | **/** | **/** | **/** | **/** | **/** | **/** | **/** | **/** | **/** | **/** |
| 2.2. Are the groups comparable at baseline? | **/** | **/** | **/** | **/** | **/** | **/** | **/** | **/** | **/** | **/** | **/** | **/** | **/** |
| 2.3. Are there complete outcome data? | **/** | **/** | **/** | **/** | **/** | **/** | **/** | **/** | **/** | **/** | **/** | **/** | **/** |
| 2.4. Are outcome assessors blinded to the intervention provided? | **/** | **/** | **/** | **/** | **/** | **/** | **/** | **/** | **/** | **/** | **/** | **/** | **/** |
| 2.5 Did the participants adhere to the assigned intervention? | **/** | **/** | **/** | **/** | **/** | **/** | **/** | **/** | **/** | **/** | **/** | **/** | **/** |
| **3. NON-RANDOMIZED STUDIES** |  | | | | | | | | | | | | |
| 3.1. Participants representative of target population? | **1** | **/** | **/** | **1** | **-** | **1** | **/** | **1** | **/** | **/** | **/** | **/** | **/** |
| 3.2. Measurements appropriate to both outcome and intervention/exposure? | **1** | **/** | **/** | **1** | **1** | **1** | **/** | **1** | **/** | **/** | **/** | **/** | **/** |
| 3.3. Complete outcome data? | **1** | **/** | **/** | **1** | **1** | **1** | **/** | **1** | **/** | **/** | **/** | **/** | **/** |
| 3.4. Confounders accounted for in design and analysis? | **0** | **/** | **/** | **1** | **1** | **-** | **/** | **-** | **/** | **/** | **/** | **/** | **/** |
| 3.5. During study period, intervention administered/exposure occurred as intended? | **1** | **/** | **/** | **1** | **-** | **1** | **/** | **1** | **/** | **/** | **/** | **/** | **/** |

| **MMAT Items (4., 5.)** | **16** | **17** | **18** | **19** | **20** | **21** | **22** | **23** | **24** | **25** | **26** | **27** | **28** |
| --- | --- | --- | --- | --- | --- | --- | --- | --- | --- | --- | --- | --- | --- |
| **4. QUANTITATIVE DESCRIPTIVE STUDIES** |  | | | | | | | | | | | | |
| 4.1. Sampling strategy relevant to research question? | **/** | **/** | **/** | **/** | **/** | **/** | **/** | **/** | **/** | **/** | **/** | **1** | **/** |
| 4.2. Sample representative of target population? | **/** | **/** | **/** | **/** | **/** | **/** | **/** | **/** | **/** | **/** | **/** | **1** | **/** |
| 4.3. Measurements appropriate? | **/** | **/** | **/** | **/** | **/** | **/** | **/** | **/** | **/** | **/** | **/** | **1** | **/** |
| 4.4. Risk of nonresponse bias low? | **/** | **/** | **/** | **/** | **/** | **/** | **/** | **/** | **/** | **/** | **/** | **-** | **/** |
| 4.5. Statistical analysis appropriate to answer research question? | **/** | **/** | **/** | **/** | **/** | **/** | **/** | **/** | **/** | **/** | **/** | **1** | **/** |
| **5. MIXED METHODS STUDIES** |  | | | | | | | | | | | | |
| 5.1. Adequate rationale for using mixed methods design to address research question? | **/** | **/** | **/** | **/** | **/** | **/** | **/** | **/** | **/** | **/** | **/** | **/** | **/** |
| 5.2. Different components of study effectively integrated to answer research question? | **/** | **/** | **/** | **/** | **/** | **/** | **/** | **/** | **/** | **/** | **/** | **/** | **/** |
| 5.3. Outputs of integration of qualitative and quantitative components adequately interpreted? | **/** | **/** | **/** | **/** | **/** | **/** | **/** | **/** | **/** | **/** | **/** | **/** | **/** |
| 5.4. Divergences and inconsistencies between quantitative and qualitative results adequately addressed? | **/** | **/** | **/** | **/** | **/** | **/** | **/** | **/** | **/** | **/** | **/** | **/** | **/** |
| 5.5. Different components of study adhere to quality criteria of each tradition of methods involved? | **/** | **/** | **/** | **/** | **/** | **/** | **/** | **/** | **/** | **/** | **/** | **/** | **/** |
| **Total score** | **4** | **5** | **5** | **5** | **3** | **4** | **5** | **4** | **5** | **5** | **5** | **4** | **5** |
