# Supplementary material for: A systematic review and narrative synthesis of the research provisions under the Mental Capacity Act (2005) in England and Wales: Recruitment of adults with capacity and communication difficulties
Source: PLoS One. 2021 Sep 1;16(9):e0256697. doi: 10.1371/journal.pone.0256697 (PMC8409627; doi:10.1371/journal.pone.0256697)
Supplement: S6 Table — (DOCX) [file pone.0256697.s007.docx]

**Table 8: ASSENT statement solutions on including adults with capacity and communication difficulties in ethically-sound research**

| Recruitment | - Gatekeepers (e.g. clinicians, other healthcare professionals, care home managers etc.) are key to identifying and approaching potential participants first, engage with them. - Introductory meetings with potential participants and their familiar others (e.g. family members, support workers) are a useful way to introduce yourself and the study in a social gathering. - Follow up meetings with individuals provide useful opportunities for questions to be posed and answers to be solicited. - Opportunities to become familiar with the environment in which the research will take place may serve to inform, not only the recruitment procedure, but also future data collection. By conducting orientation visits, the researcher will be able to understand the dynamics of the environment, and commence relationship building with staff and potential participant in an informal way. In addition to supporting recruitment, this may also provide a basis for conducting capacity assessments. |
| --- | --- |
| Gatekeeper and familiar others | - Researchers need to: consider how information about their project is presented to gatekeepers as this might impact on their engagement or disengagement with the research; target different levels of information presentation for different groups of gatekeepers, e.g., information format and content might be different for a care home manager and care home staff. - Early relationship-building with gatekeepers is recommended. Typically, in positions of authority, such as managers of residential homes or day centres, they manage any perceived risks to the people using the service (e.g. residents, patients). Alternatively, the role may fall to a family member, keyworker or carer. Regardless of who is associated with access to potential participants, the researcher is advised to prioritise effective communications from the beginning to facilitate the recruitment process and to prevent exclusions. - For studies recruiting from social and health care environments, the researcher needs to be cognisant of the people who are important to the individual. Early communication with partners, relatives and friends who are close to the individual may be helpful in not only responding to capacity and communication difficulties, but also in the event of requiring consultee involvement further down the line. |
| Capacity assessment | - Whilst for research under the MCA (2005), capacity needs to be determined categorically for recruitment procedures, it should also be viewed as a spectrum that requires gradations of support for capacity to be realised in some cases. - Conducting assessments of capacity in a familiar environment to the individual may serve to alleviate any anxieties and therefore encourage the individual to perform at their true level. - Although various tools are available purporting to assess individual capacity (e.g. MacArthur Competence Assessment Tool for Clinical Research (MacCAT-CR) , capacity assessment should be carried out in the context of the research because it varies according to the complexity of information, the implications of participation and therefore the complexity of the actual decision. - Assessment could be formal or informal but should still measure the four generally accepted components of capacity for competent decision-making: understanding, retaining, reasoning and expressing a choice. - Capacity assessment should never be based on diagnosis and should be assessed or confirmed by the researcher. |
| Context for informed consent | - Recruitment should take place in a familiar environment used by the potential participant, where familiar others, (e.g. family member or carer), known to the person, can give support as required. - Time needs to be allocated for the individual to think about what participating in the study means and to discuss with trusted people as required. - Researchers should be flexible and be ready to use alternatives to the traditional method of written consent. An enhanced or consent process may be indicated to cater for a spectrum of mental capacity. - Even after gaining informed consent to participate in a study, consent needs to be monitored over time. Researchers need to watch out for signs of assent and dissent, such as verbal or non-verbal signals to indicate engagement or disengagement e.g. signs of passivity, reluctance, frustration or anxiety. - Learn about participants from relatives and carers who know the individual well. This will be useful in picking up non-verbal signals and cues more appropriately. |
| Presentation of study information | - Researchers should aim to present information about the project using a range of media that goes beyond text supported by pictorial images. Other possibilities might include film of realistic simulations of cartoon animations, PowerPoint presentations, audio files. In addition, it is recommended that different needs are catered for including understanding and expression, memory and recall, attention control, vision and hearing. - To make meaningful and relevant adaptations to the way study information is conveyed, the researcher needs to learn about the individual’s communication skillset and monitor each interaction for aversive responses indicating dissent. - By working with a group of experts-by-experience (e.g. service users, carers), researchers can ensure that information presented to potential participant is relevant and easy to understand - People are different and one size does not fit all. Researchers need to consider using multiple approaches to presenting study information. For example, presenting a full information sheet, abbreviated version and a range of accessible formats. If possible, find out how best people understand things and personalise for them. |
| Participation in ongoing decision-making | - For those lacking capacity, a personal consultee should be identified. Early engagement with familiar others will facilitate this process. If this is not possible a nominated consultee may advise on the likely wishes and feeling of the individual around participation. - Where a participant is assessed as lacking capacity in the context of the study, a consultee gives advice, but this process in itself does not obviate the need to involve the person in expressing their own wishes and feelings. A procedure is put in place to enable participation in the decision-making process on an ongoing basis whereby opportunities for self-expression are provided on a regular basis through the study period (e.g. showing the objects to be used at each data collection session) and recording their responses as evidence of ‘engaged assent’ or ‘engaged dissent’. - For those lacking capacity, the presence of people they know or familiar with may help support understanding. |
