# Supplementary material for: A systematic review and narrative synthesis of the research provisions under the Mental Capacity Act (2005) in England and Wales: Recruitment of adults with capacity and communication difficulties
Source: PLoS One. 2021 Sep 1;16(9):e0256697. doi: 10.1371/journal.pone.0256697 (PMC8409627; doi:10.1371/journal.pone.0256697)
Supplement: S1 File — Review protocol registered with PROSPERO (International prospective register of systematic reviews). (PDF) [file pone.0256697.s009.pdf]

The application of regulatory frameworks in research involving adults with communication and/or capacity difficulties in England and Wales: a systematic review and narrative synthesis

Oluseyi Florence Jimoh, Karen Bunning, Hayley Ryan, Anne Killett, Ciara Shiggins, Peter Langdon, Marcus Redley, Rob Heywood

Citation

Oluseyi Florence Jimoh, Karen Bunning, Hayley Ryan, Anne Killett, Ciara Shiggins, Peter Langdon, Marcus Redley, Rob Heywood. The application of regulatory frameworks in research involving adults with communication and/or capacity difficulties in England and Wales: a systematic review and narrative synthesis. PROSPERO 2020 CRD42020195652 Available from:
https://www.crd.york.ac.uk/prospero/display_record.php?ID=CRD42020195652

Review question

How have adults with impairments of capacity and/or communication (ICC) been recognised and accommodated in research in England and Wales since the implementation of the Mental Capacity Act?

Searches

Applied Social Sciences Index and Abstracts (ASSIA); Academic Search Complete; Cumulative Index to Nursing and Allied Health Literature (CINAHL) Complete; MEDLINE Complete; PsycArticles; PsycINFO; and ScienceDirect. Further, we will make use of forward and backward searching of eligible studies.

Types of study to be included

All study types (except regulated clinical trials and research with human tissues) where people with impairments of capacity and/or communication have been recruited as participants.

Condition or domain being studied

Application of regulatory frameworks in research involving adults with any condition associated with impairments of capacity and/or communication such as autism, stroke, acquired brain injury, dementia, mental health and intellectual disabilities.

Participants/population

Inclusion Criteria

Any research study conducted in England and/or Wales that included participants aged 16 years and above, with impairments of capacity and/or communication (e.g. autism; stroke; mental health; dementia; acquired brain injury; and intellectual disabilities).

Exclusion criteria

Research studies governed by The Medicines for Human Use (Clinical Trials) Regulations 2004 where authorisation was required by the Medicines & Healthcare products Regulatory Agency.

Research using tissue samples

Intervention(s), exposure(s)

The focus of the review will be the use and application of regulatory frameworks in accordance with the Mental Capacity Act (2005) by researchers in relation to participants with impairments of capacity and/or communication as enacted and used during their study.

Comparator(s)/control

Not applicable

Main outcome(s)

(a) A description of the range of participants with impairments of capacity and/or communication that were recruited within included studies inclusive of demographic data.

(b) The identification and synthesis of the procedures used to recruit these participants.

(c) A description and synthesis of the accommodations used by researchers to support participant involvement in research.

* Measures of effect

Not applicable

Additional outcome(s)

None

* Measures of effect

Not applicable

Data extraction (selection and coding)

1. Articles resulting from the database search will be exported to a reference management tool (e.g. EndNote version 8) and duplicates removed.

2. After the removal of duplicates, two reviewers will independently screen titles and abstracts against the eligibility criteria.

3. Papers identified will be subjected to full text screening by two independent reviewers.

4. Any disagreement between screeners will be resolved through discussion with a third researcher until agreement is reached.

5. A standardised form will be used to extract data from the included studies for assessment of quality and evidence synthesis. Where possible the following data will be extracted: Participant characteristics e.g. diagnosis of population group, number of eligible participants, inclusion/exclusion criteria, participant information format, capacity assessment methods, informed consent method, a descriptive of accommodations made for participants who lack capacity.

Risk of bias (quality) assessment

A quality appraisal tool to score qualitative, quantitative and mixed method research using the same scale will be used (e.g. Mixed Methods Appraisal Tool (MMAT; Hong et. Al., 2018). We will focus on: selection bias, study design, data collection methods and sample size.

Strategy for data synthesis

Because of the anticipated diversity in the included studies regarding sample characteristics, settings and interventions, narrative synthesis will be completed (Popay 2006). Using a textual approach, this will start with a clear, descriptive summary of the included studies in tabular format, detailing the authors and year; study design; sample size; sample criteria - inclusion and exclusion; informed consent procedure (including assent where present). Relationships within and between the studies will be inspected, including identification of characteristics that vary across the studies and using visual methods (e.g. conceptual mapping) to group those studies that share similar characteristics.

Analysis of subgroups or subsets

None

Contact details for further information

Oluseyi Florence Jimoh
O.Jimoh@uea.ac.uk

Organisational affiliation of the review
University of East Anglia
http://www.uea.ac.uk/

Review team members and their organisational affiliations
Dr Oluseyi Florence Jimoh. University of East Anglia
Dr Karen Bunning. University of East Anglia
Ms Hayley Ryan. University of East Anglia
Anne Killett. University of East Anglia
Ms Ciara Shiggins. La Trobe University, Melbourne
Dr Peter Langdon. University of Warwick
Dr Marcus Redley. University of East Anglia
Professor Rob Heywood. University of East Anglia

Type and method of review
Methodology, Narrative synthesis, Systematic review

Anticipated or actual start date
09 December 2019

Anticipated completion date
30 October 2020

Funding sources/sponsors
Nuffield Foundation

Grant number(s)

State the funder, grant or award number and the date of award

OSAP/43239

Conflicts of interest

Language
English

Country
England

Stage of review
Review Ongoing

Subject index terms status
Subject indexing assigned by CRD

Subject index terms
Adult; Communication; England; Humans; Narration; Wales

Date of registration in PROSPERO
28 July 2020

Date of first submission
07 July 2020

Stage of review at time of this submission

Stage	Started	Completed
Preliminary searches	Yes	No
Piloting of the study selection process	Yes	No
Formal screening of search results against eligibility criteria	Yes	No
Data extraction	No	No
Risk of bias (quality) assessment	No	No
Data analysis	No	No

The record owner confirms that the information they have supplied for this submission is accurate and complete and they understand that deliberate provision of inaccurate information or omission of data may be construed as scientific misconduct.

The record owner confirms that they will update the status of the review when it is completed and will add publication details in due course.

Versions

28 July 2020

PROSPERO
This information has been provided by the named contact for this review. CRD has accepted this information in good faith and registered the review in PROSPERO. The registrant confirms that the information supplied for this submission is accurate and complete. CRD bears no responsibility or liability for the content of this registration record, any associated files or external websites.
