# Supplementary material for: A systematic review and narrative synthesis of the research provisions under the Mental Capacity Act (2005) in England and Wales: Recruitment of adults with capacity and communication difficulties
Source: PLoS One. 2021 Sep 1;16(9):e0256697. doi: 10.1371/journal.pone.0256697 (PMC8409627; doi:10.1371/journal.pone.0256697)
Supplement: S2 File — (DOCX) [file pone.0256697.s010.docx]

## S2 File. Full search strategies

Literature search: Applying the provisions for research under the Mental Capacity Act (2005) in England and Wales to adults with communication and/or capacity difficulties

1. **MEDLINE (PubMed)**

#14 Search: (#9) AND (#13) Filters: Journal Article, Humans, English, from 2007 – 2019,

Adolescent: 13-18 years, Adult: 19+ years

#13 Search: (#9) AND (#12)

#12 Search: (#10) OR (#11)

#11 Search: ("mental capacity") OR ("mental competency")

#10 Search: ((((("assent") OR ("autonom")) OR ("decision making")) OR

("empower")) OR ("informed consent")) OR ("ethics")

#9 Search: (#7) OR (#8)

#8 Search: ((#4) OR (#5)) OR (#6)

#7 Search: (((#1) OR (#2)) OR (#3))

#6 Search: ("stroke") OR ("aphasia")

#5 Search: (("mental illness") OR ("psychiatric disorder")) OR ("psychiatric illness")

#4 Search: "learning disab*" OR "intellectual disab*"

#3 Search: (("head injur*") OR ("brain injur*")) OR ("brain damage")

#2 Search: ((dement*) OR (dementia)) OR (alzheimer)

#1 Search: autis

**2. Applied Social Sciences Index & Abstracts (ASSIA)**

S29 s17 AND s25 Limits applied Applied Social Sciences Index & Abstracts (ASSIA) 128 Actions

S28 s17 AND s25 Limits applied Applied Social Sciences Index & Abstracts (ASSIA) 128 Actions

S27 s17 AND s25 Limits applied Applied Social Sciences Index & Abstracts (ASSIA) 128 Actions

S26 s17 AND s25 Applied Social Sciences Index & Abstracts (ASSIA) 128 Actions

S25 s18 or s19 Limits applied Applied Social Sciences Index & Abstracts (ASSIA) 305 Actions

S24 s18 or s19 Limits applied Applied Social Sciences Index & Abstracts (ASSIA) 305 Actions

S23 s18 or s19 Limits applied Applied Social Sciences Index & Abstracts (ASSIA) 5,583 Actions

S22 s18 or s19 Limits applied Applied Social Sciences Index & Abstracts (ASSIA) 5,797 Actions

S21 s18 or s19 Limits applied Applied Social Sciences Index & Abstracts (ASSIA) 114,917 Actions

S20 s18 or s19 Applied Social Sciences Index & Abstracts (ASSIA) 114,917 Actions

S19 "mental capacity" OR "mental competency" Limits applied Applied Social Sciences Index & Abstracts (ASSIA) 1,687 Actions

S18 "assent" OR "autonom*" OR "decision making" OR "empower" OR "informed consent" Limits applied Applied Social Sciences Index & Abstracts (ASSIA) 114,289 Actions

S17 autis* OR ((dement* OR "dementia" OR alzheimer*) AND PEER(yes)) OR (((head injur*) OR "brain damage" OR (brain injur*)) AND PEER(yes)) OR (("learning disab*" OR "intellectual disab*") AND PEER(yes)) OR (("mental illness" OR "mental disorder" OR "psychiatric illness" OR "psychiatric disorder") AND PEER(yes)) OR (("stroke" OR "aphasia") AND PEER(yes)) Limits applied Applied Social Sciences Index & Abstracts (ASSIA) 238 Actions

S16 autis* OR ((dement* OR "dementia" OR alzheimer*) AND PEER(yes)) OR (((head injur*) OR "brain damage" OR (brain injur*)) AND PEER(yes)) OR (("learning disab*" OR "intellectual disab*") AND PEER(yes)) OR (("mental illness" OR "mental disorder" OR "psychiatric illness" OR "psychiatric disorder") AND PEER(yes)) OR (("stroke" OR "aphasia") AND PEER(yes)) Limits applied Applied Social Sciences Index & Abstracts (ASSIA) 14 Actions

S15 autis* OR ((dement* OR "dementia" OR alzheimer*) AND PEER(yes)) OR (((head injur*) OR "brain damage" OR (brain injur*)) AND PEER(yes)) OR (("learning disab*" OR "intellectual disab*") AND PEER(yes)) OR (("mental illness" OR "mental disorder" OR "psychiatric illness" OR "psychiatric disorder") AND PEER(yes)) OR (("stroke" OR "aphasia") AND PEER(yes)) Limits applied Applied Social Sciences Index & Abstracts (ASSIA) 66 Actions

S14 autis* OR ((dement* OR "dementia" OR alzheimer*) AND PEER(yes)) OR (((head injur*) OR "brain damage" OR (brain injur*)) AND PEER(yes)) OR (("learning disab*" OR "intellectual disab*") AND PEER(yes)) OR (("mental illness" OR "mental disorder" OR "psychiatric illness" OR "psychiatric disorder") AND PEER(yes)) OR (("stroke" OR "aphasia") AND PEER(yes)) Limits applied Applied Social Sciences Index & Abstracts (ASSIA) 66 Actions

S13 autis* OR ((dement* OR "dementia" OR alzheimer*) AND PEER(yes)) OR (((head injur*) OR "brain damage" OR (brain injur*)) AND PEER(yes)) OR (("learning disab*" OR "intellectual disab*") AND PEER(yes)) OR (("mental illness" OR "mental disorder" OR "psychiatric illness" OR "psychiatric disorder") AND PEER(yes)) OR (("stroke" OR "aphasia") AND PEER(yes)) Limits applied Applied Social Sciences Index & Abstracts (ASSIA) 67 Actions

S12 autis* OR ((dement* OR "dementia" OR alzheimer*) AND PEER(yes)) OR (((head injur*) OR "brain damage" OR (brain injur*)) AND PEER(yes)) OR (("learning disab*" OR "intellectual disab*") AND PEER(yes)) OR (("mental illness" OR "mental disorder" OR "psychiatric illness" OR "psychiatric disorder") AND PEER(yes)) OR (("stroke" OR "aphasia") AND PEER(yes)) Limits applied Applied Social Sciences Index & Abstracts (ASSIA) 238 Actions

S11 autis* OR ((dement* OR "dementia" OR alzheimer*) AND PEER(yes)) OR (((head injur*) OR "brain damage" OR (brain injur*)) AND PEER(yes)) OR (("learning disab*" OR "intellectual disab*") AND PEER(yes)) OR (("mental illness" OR "mental disorder" OR "psychiatric illness" OR "psychiatric disorder") AND PEER(yes)) OR (("stroke" OR "aphasia") AND PEER(yes)) Limits applied Applied Social Sciences Index & Abstracts (ASSIA) 4,259 Actions

S10 autis* OR ((dement* OR "dementia" OR alzheimer*) AND PEER(yes)) OR (((head injur*) OR "brain damage" OR (brain injur*)) AND PEER(yes)) OR (("learning disab*" OR "intellectual disab*") AND PEER(yes)) OR (("mental illness" OR "mental disorder" OR "psychiatric illness" OR "psychiatric disorder") AND PEER(yes)) OR (("stroke" OR "aphasia") AND PEER(yes)) Limits applied Applied Social Sciences Index & Abstracts (ASSIA) 4,608 Actions

S9 autis* OR ((dement* OR "dementia" OR alzheimer*) AND PEER(yes)) OR (((head injur*) OR "brain damage" OR (brain injur*)) AND PEER(yes)) OR (("learning disab*" OR "intellectual disab*") AND PEER(yes)) OR (("mental illness" OR "mental disorder" OR "psychiatric illness" OR "psychiatric disorder") AND PEER(yes)) OR (("stroke" OR "aphasia") AND PEER(yes)) Limits applied Applied Social Sciences Index & Abstracts (ASSIA) 111,605 Actions

S8 autis* OR ((dement* OR "dementia" OR alzheimer*) AND PEER(yes)) OR (((head injur*) OR "brain damage" OR (brain injur*)) AND PEER(yes)) OR (("learning disab*" OR "intellectual disab*") AND PEER(yes)) OR (("mental illness" OR "mental disorder" OR "psychiatric illness" OR "psychiatric disorder") AND PEER(yes)) OR (("stroke" OR "aphasia") AND PEER(yes)) Applied Social Sciences Index & Abstracts (ASSIA) 112,450 Actions

S7 "stroke" OR "aphasia" Limits applied Applied Social Sciences Index & Abstracts (ASSIA) 17,286 Actions

S6 "mental illness" OR "mental disorder" OR "psychiatric illness" OR "psychiatric disorder" Limits applied Applied Social Sciences Index & Abstracts (ASSIA) 38,571 Actions

S5 "learning disab*" OR "intellectual disab*" Limits applied Applied Social Sciences Index & Abstracts (ASSIA) 20,558 Actions

S4 (head injur*) OR "brain damage" OR (brain injur*) Limits applied Applied Social Sciences Index & Abstracts (ASSIA) 17,147 Actions

S3 dement* OR "dementia" OR alzheimer* Limits applied Applied Social Sciences Index & Abstracts (ASSIA) 22,699 Actions

S2 dement* Applied Social Sciences Index & Abstracts (ASSIA) 21,114 Actions

S1 autis* Applied Social Sciences Index & Abstracts (ASSIA) 23,846 Actions

**3. CINAHL Complete**

| S16 | S11 AND S15 | Expanders - Apply related words; Apply equivalent subjects Search modes - Find all my search terms | 111 |
| --- | --- | --- | --- |
| S15 | S12 OR S13 | Limiters - Published Date: 20190101-20191231; English Language; Peer Reviewed; Geographic Subset: UK & Ireland; Language: English; Age Groups: Adolescent: 13-18 years, All Adult Expanders - Apply related words; Apply equivalent subjects Search modes - Find all my search terms | 1,710 |
| S14 | S12 OR S13 | Expanders - Apply related words; Apply equivalent subjects Search modes - Find all my search terms | 290,074 |
| S13 | (mental capacity) OR (mental competency) | Expanders - Apply related words; Apply equivalent subjects Search modes - Find all my search terms | 13,962 |
| S12 | (assent) OR (autonom*) OR (decision making) OR (empower) OR (informed consent) OR (ethics) | Expanders - Apply related words; Apply equivalent subjects Search modes - Find all my search terms | 278,998 |
| S11 | S9 OR S10 | Expanders - Apply related words; Apply equivalent subjects Search modes - Find all my search terms | 1,370 |
| S10 | S6 OR S7 OR S8 | Expanders - Apply related words; Apply equivalent subjects Search modes - Find all my search terms | 977 |
| S9 | S3 OR S4 OR S5 | Expanders - Apply related words; Apply equivalent subjects Search modes - Find all my search terms | 501 |
| S8 | (stroke) OR (aphasia) | Limiters - Published Date: 20190101-20191231; English Language; Peer Reviewed; Exclude MEDLINE records; Geographic Subset: UK & Ireland; Language: English; Age Groups: Adolescent: 13-18 years, All Adult Expanders - Apply related words; Apply equivalent subjects Search modes - Find all my search terms | 294 |
| S7 | (mental illness*) OR (mental disorder*) OR (psychiatric illness*) OR (psychiatric disorder*) | Limiters - Published Date: 20190101-20191231; English Language; Peer Reviewed; Exclude MEDLINE records; Geographic Subset: UK & Ireland; Language: English; Age Groups: Adolescent: 13-18 years, All Adult Expanders - Apply related words; Apply equivalent subjects Search modes - Find all my search terms | 499 |
| S6 | (learning disab*) OR (intellectual disab*) | Limiters - Published Date: 20190101-20191231; English Language; Peer Reviewed; Exclude MEDLINE records; Geographic Subset: UK & Ireland; Language: English; Age Groups: Adolescent: 13-18 years, All Adult Expanders - Apply related words; Apply equivalent subjects Search modes - Find all my search terms | 222 |
| S5 | (head injur*) OR (brain injur*) OR (brain damage) | Limiters - Published Date: 20190101-20191231; English Language; Peer Reviewed; Exclude MEDLINE records; Geographic Subset: UK & Ireland; Language: English; Age Groups: Adolescent: 13-18 years, All Adult Expanders - Apply related words; Apply equivalent subjects Search modes - Find all my search terms | 128 |
| S4 | (dement*) OR (dementia) OR (alzheimer*) | Limiters - Published Date: 20190101-20191231; English Language; Peer Reviewed; Exclude MEDLINE records; Geographic Subset: UK & Ireland; Language: English; Age Groups: Adolescent: 13-18 years, All Adult Expanders - Apply related words; Apply equivalent subjects Search modes - Find all my search terms | 232 |
| S3 | autis* | Limiters - Published Date: 20190101-20191231; English Language; Peer Reviewed; Exclude MEDLINE records; Geographic Subset: UK & Ireland; Language: English; Age Groups: Adolescent: 13-18 years, All Adult Expanders - Apply related words; Apply equivalent subjects Search modes - Find all my search terms | 142 |
| S2 | autis* | Limiters - Published Date: 19620101-20101231 Expanders - Apply related words; Apply equivalent subjects Search modes - Find all my search terms | 8,825 |
| S1 | autis* | Expanders - Apply related words; Apply equivalent subjects Search modes - Find all my search terms | 33,809 |

**4. PsycArticles**

S14 S9 AND S13 Expanders - Apply related words; Apply equivalent subjects Search modes - Find all my search terms 62

S13 S10 OR S11 Limiters - Published Date: 20190101- 20191231; Scholarly (Peer Reviewed) Journals; Exclude Book Reviews; Age Groups: Adolescence (13-17 yrs), Adulthood (18 yrs & older); Population Group: Human Expanders - Apply related words; Apply equivalent subjects Search modes - Find all my search terms 398

S12 S10 OR S11 Expanders - Apply related words; Apply equivalent subjects Search modes - Find all my search terms 12,028

S11 (mental capacity) OR (mental competency) Expanders - Apply related words; Apply equivalent subjects Search modes - Find all my search terms 2,490

S10 (empower*) OR (assent) OR (autonom*) OR (decision making) OR (informed consent) Expanders - Apply related words; Apply equivalent subjects Search modes - Find all my search terms 9.767

S9 S7 OR S8 Expanders - Apply related words; Apply equivalent subjects Search modes - Find all my search terms 688

S8 S4 OR S5 OR S6 Expanders - Apply related words; Apply equivalent subjects Search modes - Find all my search terms 585

S7 S1 OR S2 OR S3 Expanders - Apply related words; Apply equivalent subjects Search modes - Find all my search terms 155

S6 (stroke) OR (aphasia) Limiters - Published Date: 20190101- 20191131; Scholarly (Peer Reviewed) Journals; Exclude Book Reviews; Age Groups: Adolescence Adulthood (18 yrs & older); Population Group: Human; Document Type: Journal Article Expanders - Apply related words; Apply equivalent subjects Search modes - Find all my search terms (13-17 yrs), Adulthood (18 yrs & older); Population Group: Human; Document Type: Journal Article Expanders - Apply related words; Apply equivalent subjects Search modes - Find all my search terms 17

S5 (mental illness*) OR (mental disorder*) OR (psychiatric illness*) OR (psychiatric disorder*) Limiters - Published Date: 20190101- 20191131; Scholarly (Peer Reviewed) Journals; Exclude Book Reviews; Age Groups: Adolescence (13-17 yrs), Adulthood (18 yrs & older); Population Group: Human; Document Type: Journal Article Expanders - Apply related words; Apply equivalent subjects Search modes - Find all my search terms 553

S4 (learning disab*) OR (intellectual disab*) Limiters - Published Date: 20190101- 20191131; Scholarly (Peer Reviewed) Journals; Exclude Book Reviews; Age Groups: Adolescence (13-17 yrs), Adulthood (18 yrs & older); Population Group: Human; Document Type: Journal Article Expanders - Apply related words; Apply equivalent subjects Search modes - Find all my search terms 20

S3 (head injur*) OR (brain injur*) OR (brain damage) Limiters - Published Date: 20190101- 20191131; Scholarly (Peer Reviewed) Journals; Exclude Book Reviews; Age Groups: Adolescence (13-17 yrs), Adulthood (18 yrs & older); Population Group: Human; Document Type: Journal Article Expanders - Apply related words; Apply equivalent subjects Search modes - Find all my search terms 94

S2 (dement*) OR (dementia) OR (alzheimer*) Limiters - Published Date: 20190101- 20191131; Scholarly (Peer Reviewed) Journals; Exclude Book Reviews; Age Groups: Adolescence (13-17 yrs), Adulthood (18 yrs & older); Population Group: Human; Document Type: Journal Article Expanders - Apply related words; Apply equivalent subjects Search modes - Find all my search terms 32

S1 autis* Limiters - Published Date: 20190101- 20191131; Scholarly (Peer Reviewed) Journals; Exclude Book Reviews; Age Groups: Adolescence (13-17 yrs) Adulthood (18 yrs & older); Population Group: Human; Document Type: Journal Article Expanders - Apply related words; Apply equivalent subjects Search modes - Find all my search terms 48

1. **PschINFO**

| S14 | S9 AND S13 | Expanders - Apply related words; Apply equivalent subjects Search modes - Find all my search terms | 979 |
| --- | --- | --- | --- |
| S13 | S10 OR S11 | Limiters - Published Date: 20190101-20191231; Peer Reviewed; English; Language: English; Age Groups: Adolescence (13-17 yrs), Adulthood (18 yrs & older); Population Group: Human; Exclude Dissertations Expanders - Apply related words; Apply equivalent subjects Search modes - Find all my search terms | 4,464 |
| S12 | S10 OR S11 | Expanders - Apply related words; Apply equivalent subjects Search modes - Find all my search terms | 276,377 |
| S11 | (mental capacity) OR (mental competency) | Expanders - Apply related words; Apply equivalent subjects Search modes - Find all my search terms | 51,902 |
| S10 | (assent) OR (autonom*) OR (decision making) OR (empower) OR (informed consent) | Expanders - Apply related words; Apply equivalent subjects Search modes - Find all my search terms | 230,171 |
| S9 | S7 OR S8 | Expanders - Apply related words; Apply equivalent subjects Search modes - Find all my search terms | 14,267 |
| S8 | S4 OR S5 OR S6 | Expanders - Apply related words; Apply equivalent subjects Search modes - Find all my search terms | 10,404 |
| S7 | S1 OR S2 OR S3 | Expanders - Apply related words; Apply equivalent subjects Search modes - Find all my search terms | 6,356 |
| S6 | (stroke) OR (aphasia) | Limiters - Published Date: 20190101-20191231; Peer Reviewed; Publication Type: Peer Reviewed Journal; English; Language: English; Age Groups: Adolescence (13-17 yrs), Adulthood (18 yrs & older); Population Group: Human; Exclude Dissertations Expanders - Apply related words; Apply equivalent subjects Search modes - Find all my search terms | 974 |
| S5 | (mental illness*) OR ( mental disorder*) OR (Psychiatric illness*) OR (psychiatric disorder*) | Limiters - Published Date: 20190101-20191231; Peer Reviewed; Publication Type: Peer Reviewed Journal; English; Language: English; Age Groups: Adolescence (13-17 yrs), Adulthood (18 yrs & older); Population Group: Human; Exclude Dissertations Expanders - Apply related words; Apply equivalent subjects Search modes - Find all my search terms | 9,238 |
| S4 | (learning disab*) OR (intellectual disab*) | Limiters - Published Date: 20190101-20191231; Peer Reviewed; Publication Type: Peer Reviewed Journal; English; Language: English; Age Groups: Adolescence (13-17 yrs), Adulthood (18 yrs & older); Population Group: Human; Exclude Dissertations Expanders - Apply related words; Apply equivalent subjects Search modes - Find all my search terms | 662 |
| S3 | (head injur*) OR (brain damage) OR (brain injur*) | Limiters - Published Date: 20190101-20191231; Peer Reviewed; Publication Type: Peer Reviewed Journal; English; Language: English; Age Groups: Adolescence (13-17 yrs), Adulthood (18 yrs & older); Population Group: Human; Exclude Dissertations Expanders - Apply related words; Apply equivalent subjects Search modes - Find all my search terms | 3,970 |
| S2 | (dement*) OR (dementia) OR (alzheimer*) | Limiters - Published Date: 20190101-20191231; Peer Reviewed; Publication Type: Peer Reviewed Journal; English; Language: English; Age Groups: Adolescence (13-17 yrs), Adulthood (18 yrs & older); Population Group: Human; Exclude Dissertations Expanders - Apply related words; Apply equivalent subjects Search modes - Find all my search terms | 2,234 |
| S1 | autis* | Limiters - Published Date: 20190101-20191231; Peer Reviewed; Publication Type: Peer Reviewed Journal; English; Language: English; Age Groups: Adolescence (13-17 yrs), Adulthood (18 yrs & older); Population Group: Human; Exclude Dissertations Expanders - Apply related words; Apply equivalent subjects Search modes - Find all my search terms | 1,592 |

1. **Academic Search Complete**

| S31 | S9 AND S10 | Limiters - Scholarly (Peer Reviewed) Journals; Published Date: 20190101-20191231; Document Type: Article; Language: English Expanders - Apply related words; Apply equivalent subjects Search modes - Find all my search terms | 336 |
| --- | --- | --- | --- |
| S30 | S16 OR S23 OR S24 OR S25 OR S26 OR S27 | Expanders - Apply related words; Apply equivalent subjects Search modes - Find all my search terms | 2,781,490 |
| S29 | "mental capacity" OR "mental competency" | Expanders - Apply related words; Apply equivalent subjects Search modes - Find all my search terms | Display |
| S28 | "ASSENT" OR "informed consent" OR "autonomy" OR "decision making" OR "empower" | Expanders - Apply related words; Apply equivalent subjects Search modes - Find all my search terms | Display |
| S27 | "stroke" OR "aphasia" OR "communication disorder*" OR "communication impairment*" | Limiters - Scholarly (Peer Reviewed) Journals; Published Date: 20180101-20191231; Document Type: Article; Language: English Expanders - Apply related words; Apply equivalent subjects Search modes - Find all my search terms | Display |
| S26 | "mental illness" OR "psychiatric disorder*" OR "psychiatric illness" OR "mental disorder*" | Limiters - Scholarly (Peer Reviewed) Journals; Published Date: 20180101-20191231; Document Type: Article; Language: English Expanders - Apply related words; Apply equivalent subjects Search modes - Find all my search terms | Display |
| S25 | "head injur*" OR "head injury" OR "brain injury" OR "brain damage" | Limiters - Scholarly (Peer Reviewed) Journals; Published Date: 20180101-20191231; Document Type: Article; Language: English Expanders - Apply related words; Apply equivalent subjects Search modes - Find all my search terms | Display |
| S24 | dement* OR dementia OR alzheimers | Limiters - Scholarly (Peer Reviewed) Journals; Published Date: 20180101-20191231; Document Type: Article; Language: English Expanders - Apply related words; Apply equivalent subjects Search modes - Find all my search terms | Display |
| S23 |  | Limiters - Scholarly (Peer Reviewed) Journals; Published Date: 20180101-20191231; Document Type: Article; Language: English Expanders - Apply related words; Apply equivalent subjects Search modes - Find all my search terms | Display |
| S22 | S30 AND S17 | Limiters - Scholarly (Peer Reviewed) Journals; Published Date: 20190101-20191231; Document Type: Article; Language: English Expanders - Apply related words; Apply equivalent subjects Search modes - Find all my search terms | Display |
| S21 | S30 AND S17 | Limiters - Scholarly (Peer Reviewed) Journals; Published Date: 20180101-20191231; Document Type: Article; Language: English Expanders - Apply related words; Apply equivalent subjects Search modes - Find all my search terms | Display |
| S20 | S30 AND S17 | Limiters - Published Date: 20180101-20191231; Document Type: Article; Language: English Expanders - Apply related words; Apply equivalent subjects Search modes - Find all my search terms | Display |
| S19 | S30 AND S17 | Limiters - Published Date: 20190101-20191231 Expanders - Apply related words; Apply equivalent subjects Search modes - Find all my search terms | Display |
| S18 | S30 AND S17 | Expanders - Apply related words; Apply equivalent subjects Search modes - Find all my search terms | Display |
| S17 | S28 OR S29 | Expanders - Apply related words; Apply equivalent subjects Search modes - Find all my search terms | Display |
| S16 |  | Limiters - Published Date: 20180101-20191231; Document Type: Article; Language: English Expanders - Apply related words; Apply equivalent subjects Search modes - Find all my search terms | Display |
| S15 | S9 AND S10 | Limiters - Scholarly (Peer Reviewed) Journals; Published Date: 20190101-20191231; Document Type: Article; Language: English Expanders - Apply related words; Apply equivalent subjects Search modes - Find all my search terms | 21,398 |
| S14 | S9 AND S10 | Limiters - Scholarly (Peer Reviewed) Journals; Published Date: 20180101-20191231; Document Type: Article; Language: English Expanders - Apply related words; Apply equivalent subjects Search modes - Find all my search terms | 40,834 |
| S13 | S9 AND S10 | Limiters - Published Date: 20180101-20191231; Document Type: Article; Language: English Expanders - Apply related words; Apply equivalent subjects Search modes - Find all my search terms | 43,517 |
| S12 | S9 AND S10 | Limiters - Published Date: 20190101-20191231 Expanders - Apply related words; Apply equivalent subjects Search modes - Find all my search terms | 22,652 |
| S11 | S9 AND S10 | Expanders - Apply related words; Apply equivalent subjects Search modes - Find all my search terms | 43,517 |
| S10 | S7 OR S8 | Expanders - Apply related words; Apply equivalent subjects Search modes - Find all my search terms | 362,069 |
| S9 | S1 OR S2 OR S3 OR S4 OR S5 OR S6 | Expanders - Apply related words; Apply equivalent subjects Search modes - Find all my search terms | 2,781,490 |
| S8 | "mental capacity" OR "mental competency" | Expanders - Apply related words; Apply equivalent subjects Search modes - Find all my search terms | 8,349 |
| S7 | "ASSENT" OR "informed consent" OR "autonomy" OR "decision making" OR "empower" | Expanders - Apply related words; Apply equivalent subjects Search modes - Find all my search terms | 354,596 |
| S6 | "stroke" OR "aphasia" OR "communication disorder*" OR "communication impairment*" | Limiters - Scholarly (Peer Reviewed) Journals; Published Date: 20180101-20191231; Document Type: Article; Language: English Expanders - Apply related words; Apply equivalent subjects Search modes - Find all my search terms | 16,658 |
| S5 | "mental illness" OR "psychiatric disorder*" OR "psychiatric illness" OR "mental disorder*" | Limiters - Scholarly (Peer Reviewed) Journals; Published Date: 20180101-20191231; Document Type: Article; Language: English Expanders - Apply related words; Apply equivalent subjects Search modes - Find all my search terms | 13,445 |
| S4 | "head injur*" OR "head injury" OR "brain injury" OR "brain damage" | Limiters - Scholarly (Peer Reviewed) Journals; Published Date: 20180101-20191231; Document Type: Article; Language: English Expanders - Apply related words; Apply equivalent subjects Search modes - Find all my search terms | 6,669 |
| S3 | dement* OR dementia OR alzheimers | Limiters - Scholarly (Peer Reviewed) Journals; Published Date: 20180101-20191231; Document Type: Article; Language: English Expanders - Apply related words; Apply equivalent subjects Search modes - Find all my search terms | 14,745 |
| S2 |  | Limiters - Scholarly (Peer Reviewed) Journals; Published Date: 20180101-20191231; Document Type: Article; Language: English Expanders - Apply related words; Apply equivalent subjects Search modes - Find all my search terms | 2,374,626 |
| S1 |  | Limiters - Published Date: 20180101-20191231; Document Type: Article; Language: English Expanders - Apply related words; Apply equivalent subjects Search modes - Find all my search terms | 2,781,490 |

1. **ScienceDirect**

| autis* AND "mental capacity" |
| --- |
| autis* AND capacity |
| autis* AND "informed consent" |
| autis* AND "decision mak*" |
| autis* AND communicat* |
| autis* AND assent |
| autis* AND dissent |
| autis* AND inclu* |
| autis AND exclu* |
| dement* AND "mental capacity" |
| dement* AND capacity |
| dement* AND "informed consent" |
| dement* AND "decision mak*" |
| dement* AND communicat* |
| dement* AND assent |
| dement* AND dissent |
| dement* AND inclu* |
| dement AND exclu* |
| "brain injur*" AND "mental capacity" |
| "brain injur*" AND capacity |
| "brain injur*" AND "informed consent" |
| "brain injur*" AND "decision mak*" |
| "brain injur*" AND communicat* |
| "brain injur*" AND assent |
| "brain injur*" AND dissent |
| "brain injur*" AND inclu* |
| "brain injur*" AND exclu* |
| "head injur*" AND "mental capacity" |
| "head injur*"AND capacity |
| "head injur*" AND "informed consent" |
| "head injur*" AND "decision mak*" |
| "head injur*" AND communicat* |
| "head injur*" AND assent |
| "head injur*" AND dissent |
| "head injur*" AND inclu* |
| "head injur*" AND exclu* |
| "mental health" AND "mental capacity" |
| "mental health" AND capacity |
| "mental health" AND "informed consent" |
| "mental health" AND "decision mak*" |
| "mental health" AND communicat* |
| "mental health" AND assent |
| "mental health" AND dissent |
| "mental health" AND inclu* |
| "mental health" AND exclu* |
| "mental illness" AND "mental capacity" |
| "mental illness" AND capacity |
| "mental illness" AND "informed consent" |
| "mental illness" AND "decision mak*" |
| "mental illness" AND communicat* |
| "mental illness" AND assent |
| "mental illness" AND dissent |
| "mental illness" AND inclu* |
| "mental illness" AND exclu* |
| "psychiatric disorder" AND "mental capacity" |
| "psychiatric disorder" AND capacity |
| "psychiatric disorder" AND "informed consent" |
| "psychiatric disorder" AND "decision mak*" |
| "psychiatric disorder" AND communicat* |
| "psychiatric disorder" AND assent |
| "psychiatric disorder" AND dissent |
| "psychiatric disorder" AND inclu* |
| "psychiatric disorder" AND exclu* |
| "psychiatric illness" AND "mental capacity" |
| "psychiatric illness" AND capacity |
| "psychiatric illness" AND "informed consent" |
| "psychiatric illness" AND "decision mak*" |
| "psychiatric illness" AND communicat* |
| "psychiatric illness" AND assent |
| "psychiatric illness" AND dissent |
| "psychiatric illness" AND inclu* |
| "psychiatric illness" AND exclu* |
| "aphasia" AND "mental capacity" |
| "aphasia" AND capacity |
| "aphasia" AND "informed consent" |
| "aphasia" AND "decision mak*" |
| "aphasia" AND communicat* |
| "aphasia" AND assent |
| "aphasia" AND dissent |
| "aphasia" AND inclu* |
| "aphasia" AND exclu* |
| "stroke" AND "mental capacity" |
| "stroke" AND capacity |
| "stroke" AND "informed consent" |
| "stroke" AND "decision mak*" |
| "stroke" AND communicat* |
| "stroke" AND assent |
| "stroke" AND dissent |
| "stroke" AND inclu* |
| "stroke" AND exclu* |
| "intellectual disab*" AND "mental capacity" |
| "intellectual disab*" AND capacity |
| "intellectual disab*" AND "informed consent" |
| "intellectual disab*" AND "decision mak*" |
| "intellectual disab*" AND communicat* |
| "intellectual disab*" AND assent |
| "intellectual disab*" AND dissent |
| "intellectual disab*" AND inclu* |
| "intellectual disab*" AND exclu* |
| "learning disab*" AND "mental capacity" |
| "learning disab*" AND capacity |
| "learning disab*" AND "informed consent" |
| "learning disab*" AND "decision mak*" |
| "learning disab*" AND communicat* |
| "learning disab*" AND assent |
| "learning disab*" AND dissent |
| "learning disab*" AND inclu* |
| "learning disab*" AND exclu* |
