# Supplementary material for: A systematic review and narrative synthesis of the research provisions under the Mental Capacity Act (2005) in England and Wales: Recruitment of adults with capacity and communication difficulties
Source: PLoS One. 2021 Sep 1;16(9):e0256697. doi: 10.1371/journal.pone.0256697 (PMC8409627; doi:10.1371/journal.pone.0256697)
Supplement: Attachment — Submitted filename: Response to Reviewers Jimoh et al.docx [file pone.0256697.s011.docx]

Oluseyi F Jimoh, PhD

Senior Research Associate,

School of Health Sciences

University of East Anglia

Norwich Research Park

Norwich NR4 7TJ

01603593584

O.Jimoh@uea.ac.uk

24^th^ June 2021

Dr Emily Chenette,

Editor-in-Chief

PLOS ONE

Dear Dr Emily Chenette,

Thank you for recommending our paper for publication with major revision.

We have tried our best to address all the issues raised. Please see our response to comments below.

**Reviewer #1**

**Reviewer 1:** I think this is a very systematic review, it is important to evaluate how consent is obtained in vulnerable populations. It is also clear that participants who could be able to take part in research should not be excluded. Some areas of the manuscript needed some further clarification/justification in order to improve it.

**Authors’ response**: Thank you for your comments on our review. We have tried out best to address the issue raised.

**Specific comments**

1. Consider revising the title to “A systematic review and narrative synthesis of the provisions used to recruit adults with capacity and communication difficulties under the mental capacity act (2005) in England and Wales”

**Authors’ response**: Thank you for your suggestion which we have adopted. We have broken it up with a colon to make it easier to follow. The title now reads:

“A systematic review and narrative synthesis of the research provisions under the Mental Capacity Act (2005) in England and Wales: Recruitment of adults with capacity and communication difficulties” (Page 1, lines 1 & 2).

2. Consider revising the first sentence in the abstract to this: “The Mental Capacity Act (2005) as well as the Code of Practise (2007), governs whether adults with capacity and communication difficulties are able to participate in research in England and Wales.”

**Authors’ response**: Thank you for suggestion. This has now been revised and now reads:

“The Mental Capacity Act (MCA, 2005) and its accompanying Code of Practise (2007), govern research participation for adults with capacity and communication difficulties in England and Wales.” (Page 2, Lines 21 & 22).

3. Last sentence of conclusion in the abstract needs to clarify why it is important that they are included – suggest adding a sentence to justify that inclusion could assist care decisions.

**Authors’ response**: Thank you for your comment.

It is beyond the scope of this review to make suggestions about care decisions as it is aimed at research procedures. We have added an explanation to justify their inclusion in research. Details below:

“If health and social interventions are to be effective for this population, they need to be included in primary research. The use of strategic adaptations and accommodations during the recruitment process, may serve to support their inclusion.” (Page 2, Lines 42 - 44).

4. The range of disorders/disease is wide – why did you select these in particular; this needs to be justified?

**Authors’ response**: Thank you for your comment.

We agree that the range of capacity and communication affecting disorder is wide. We were not interested in the whole range but have chosen to focus on six main capacity affecting conditions where we have associated communication difficulties.

We have rephrased our introductory section to reflect this (Page 3, Lines 47 - 54).

5. Why were studies involving participants aged above 16 years and not 18? This could be expanded when discussing the inclusion criteria of the studies.

**Authors’ response**: Thank you for your comment.

We included studies with participants aged 16 years and above because this is the age at which the MCA applies. The inclusion criteria have been altered to:

Participants aged 16+ years (the age at which the MCA applies), with communication and/or capacity difficulties (e.g. associated with autism; stroke; mental health; dementia; acquired brain injury; and intellectual disabilities (Page 7, Table 1).

6. Are the rules different in Scotland and Ireland? If so, this needs to be clarified as to why you only focused on studies conducted in England and Wales.

**Authors’ response:** Thank you for your comment.

The rules are slightly different in Scotland and Ireland. We have added a few sentences to the manuscript to acknowledge that there are rules in others, but the focus of our review is England and Wales. The researchers are based in England and have focused on the law guiding their own practice. Please see the addition below:

“There are separate provisions for research (CoP: Chapter 11). Different legislation is provided in other countries of the UK: the Adults with Incapacity (Scotland) Act 2000 (AWIA); the Mental Capacity Act (Northern Ireland) (2016). In Ireland, it is the Assisted Decision Making (Capacity) Act 2015. However, the current review pertains to the Mental Capacity Act (2005) in England and Wales.”. (Page 3, Lines 62-65).

7. Is 28 studies sufficient to conduct a systematic review? How many papers were given an MMAT score of 3 or less? Does excluding the lower scoring publications change the narrative?

**Authors’ response:** Thank you for your observation.

Yes, 28 studies are enough to conduct a systematic review.

The 28 studies are the outcome of the systematic search of the literature. There are no present number of studies that can be included. It all depends on how many studies fit the inclusion criteria.

Of all 28 studies, 24 were high quality, two were moderate (score 3), one scored 2. Excluding the lower scoring publications (n=3), does not change the narrative of the review.

8. Since you were looking at studies carried out in Wales – why was the inclusion criteria English only? Did some of the other studies mention that Welsh wasn’t the other languages spoken?

**Authors’ response**: Thank you for your observation.

We considered that since our focus was on the implementation of the Mental Capacity Act in England and Wales, most articles will be reported in English Language. Moreover, most articles written in Welsh will usually have an English version. In addition, we did not have a Welsh speaker on the research team which limited our ability to take on any Welsh article which has no translation. It is possible that we missed out on some of those. This has now been acknowledged as a weakness. See below:

“By limiting publication language to only English, we might have missed out on research findings reported in Welsh, the other official language apart from English in Wales.” (Page 17/18, Lines 373-375).

9. Fig 1 says 29 studies included but 28 in the abstract?

**Authors’ response:** Thank you for the observation. This is a typo and has now been corrected. (see Figure 1, page 9).

10. In discussion, clarify which strategy or strategies would best be used to allow people with reduced capacity to consent. This is a little implicit.

**Authors’ response:** Thank you for your suggestion.

The following has now been added to the discussion section:

“Recruitment procedures targeting individuals with CCD need to include deliberate measures to achieve the easiest cognitive load possible within the required research framework (1). Researchers need to be cognisant of the range of strategies and accommodations that can be used to support autonomous decision-making by engaging with the evidence on augmentation and alternative communication methods (2). This includes the use of picture, simple text, object of reference and supported conversation (3). In addition, consideration should be given to the individual need of each participant, tailoring accommodations to their preferred way of engaging with researchers (4)”. (Page 15, Lines 324-330).

**Reviewer #2**

1. In the abstract total studies included is 28 but in flow diagram 29

**Authors’ response:** Thank you for spotting that. Apologies, it is a typo. There are 28 studies and has now been corrected. See figure 1: PRISMA flow diagram of studies included. (Page 9, Figure 1).

2. P3/L48 CCD but P 4/L92 CDD in S1 ICC. Please be consistent.

**Authors’ response:** Thank you for noticing the discrepancy.

The correct acronym is CCD. It stands for Capacity and Communication difficulties. CDD in S1 ICC is in reference to our published protocol on Prospero (A supplementary document for the current manuscript), it was not used in the current manuscript.

In the earlier stages of our project, we used the term “adults with impairments of capacity and/or communication (ICC)”, as reflected in our protocol. This was later refined to “adults with capacity and communication difficulties” to make the term easier for our stakeholders.

We have now included a sentence in the methods section of our review to reflect the change of term. The sentence reads:

“In the protocol, we used the term “adults with impairments of capacity and/or communication (ICC)”. This has been refined and modified through our interactions with our stakeholders to “adults with capacity and communication difficulties”. (See Page 5/6, Lines: 124-126).

3. Only 2/28 studies included are mix-method, why MMAT as the main quality appraisal?

**Authors’ response:** Thank you for your observation.

We used the Mixed Methods Appraisal Tool (MMAT) for consistency in assessing the quality of all included studies.

The following has now been added to the ‘Data extraction and quality assessment’ section of the “Methods” to justify our use of their tool:

“The Mixed Methods Appraisal Tool (MMAT) (5), for concurrent critical appraisal of quantitative, qualitative and mixed-methods primary research was applied (6). The MMAT has established content validity, it has been piloted across all methodologies; quantitative, qualitative and mixed methods research designs (6,7). Compared with other tools, the MMAT includes specific criteria for appraising mixed methods studies. While critical appraisal tools are more widely available for quantitative and qualitative research, there has not been consensus on quality criteria for mixed methods research (8)”. (Page 8, lines: 159-164).

4. Page 17: line 226: Capacity assessment procedures were typically not reported in detail. Please change this style of writing.

**Authors’ response:** Thank you for your comment. We have modified this to:

“Capacity assessment procedures were not reported in detail in many studies.” (P12, Line 259).

**Further comments (Academic editor)**

1. The introduction was not well articulated, the research gap and significant of the study are not clear.

**Authors’ response:** Thank you for your comment.

The literature review has now been updated, research gap identified, and the significance improved. Please see the introductory section.

2. Theoretical part for on CCD is missing and little available. We recommend authors to add some theoretical ideas at introduction and also discussion.

**Authors’ response:** Thank you for your suggestion.

The introduction and discussion sections have been updated with some theoretical ideas.

Introduction:

“For the purposes of research, there is the presumption of capacity unless there is a reason to believe that a person lacks capacity (CoP 2007). Before deciding that someone lacks capacity, the CoP (2007) recommends the provision of relevant information, communicated in the most appropriate way (4). Whilst practical details are not given, there is general encouragement for presenting project information to suit the processing capabilities of potential participants. For example, support for the person’s understanding of what research participation entails might include: information sheets rendered in simple language with or without pictorial support; a simulated data collection procedure shown on video; questions and answer opportunities in conversations about a project; and use of manual sign and gesture to augment meanings (2–4,9). Relevance theory (1) argues that people find it easier to engage with and understand information that is most relevant to them and requires the least cognitive effort. The form of the message interacts with the person’s cognitive abilities, prior experience and underlying knowledge. On this latter point, the person’s familiarity with the subject matter contributes to their perception of possible cognitive gain, which in turn optimises the potential relevance of information to them (1). This asserts the importance of addressing the information-processing needs of the target population for successful recruitment to studies, particularly where CCDs are present.” (P3/4, Lines 70-84).

Discussion

A range of strategies was used by researchers to support the accessibility of research information for those with CCD. “This is consistent with relevance theory (1), as understanding of research information will be based on the cognitive load of each strategy”.

Previous studies have shown that ‘aphasia-friendly’ study information was preferred by the aphasic participants (10) and led to 11.2% increase in their understanding (11). “This resonates the underlying premise of relevance theory that successful engagement with information requires the least cognitive load (1).”

3. Since the majority (85.7%) of the studies included were quantitative, we suggest authors to use a PICO frame.

**Authors’ response:** Thank you for your comment.

The PICO framework is better suited for therapy questions. We have used it as much as possible in Table 1, to describe the eligibility criteria for included studies. please see Table 1 for details (page 7).

4. References We scan all references. Some of them are incomplete. Reference 3/ 4 / 53 /78 Therefore, I recommend this paper for publication with major revision.

**Authors’ response:** Thank you for your observation. The reference identified have now been revised.

We hope we have been able to respond to all your queries appropriately.

Thank you for your consideration.

Authors: Oluseyi F. Jimoh and colleagues

**References**

1.	Sperber D, Wilson D. Relevance: Communication and Cognition. 2nd Editio. Oxford: Blackwell Publishing Inc; 1986. 1995 p.

2.	Beukelman D, Light J. Augmentative & Alternative Communication: Supporting Children and Adults with Complex Communication Needs. 5th editio. London, UK: Blackwell Publishing Inc; 2020.

3.	MENCAP. Accessible Information Standard [Internet]. Available from: https://www.mencap.org.uk/accessible-information-standard

4.	Department for Constitutional Affairs. Mental Capacity Act 2005 Code of Practice [Internet]. London: The Stationary Office. 2007. p. 1–301. Available from: https://assets.publishing.service.gov.uk/government/uploads/system/uploads/attachment_data/file/497253/Mental-capacity-act-code-of-practice.pdf

5.	Hong Q, Fàbregues S, Bartlett G, Boardman F, Cargo M, Dagenais P, et al. (10 more authors) (2018) The Mixed Methods Appraisal Tool (MMAT) version 2018 for information professionals and researchers. Educ Inf. 2018;34(4):285–91.

6.	Pace R, Pluye P, Bartlett G, Macaulay A, Salsberg J, Jagosh J, et al. Testing the reliability and efficiency of the pilot Mixed Methods Appraisal Tool (MMAT) for systematic mixed studies review. Int J Nurs Stud J. 2012;49(1):Epub 2011 Aug 10. PMID: 21835406.

7.	Souto R, Khanassov V, Hong Q, Bush P, Vedel I, Pluye P. Systematic mixed studies reviews: updating results on the reliability and efficiency of the mixed methods appraisal tool. Int J Nurs Stud. 2015;52:500–1.

8.	O’Cathain A, Murphy E, Nicholl J. The quality of mixed methods studies in health services research. J Health Serv Res Policy. 2008;92-8.(2):92–8.

9.	Department of Health. Making written information easier to understand for people with learning disabilities: Guidance for people who commission or produce Easy Read information – Revised Edition 2010. Dep Heal. 2010;1–40.

10.	Taylor J, DeMers S, Vig E, Borson S. The disappearing subject: exclusion of people with cognitive impairment and dementia from geriatrics research. J Am Geriatr Soc. 2012;60:413–9.

11.	Rose TA, Worrall LE, Hickson LM, Hoffmann TC. Aphasia friendly written health information: Content and design characteristics. Int J Speech Lang Pathol. 2011;13(4):335–47.
